# A Rapid Method of Detecting Autoantibody against FcεRIα for Chronic Spontaneous Urticaria

**DOI:** 10.1371/journal.pone.0109565

**Published:** 2014-10-15

**Authors:** Mey-Fann Lee, Tzu-Mei Lin, Szu-Wei Liu, Yi-Hsing Chen

**Affiliations:** 1 Department of Medical Research, Taichung Veterans General Hospital, Taichung, Taiwan; 2 Division of Allergy, Immunology and Rheumatology, Taichung Veterans General Hospital, Taichung, Taiwan; 3 Faculty of Medicine, National Yang-Ming University, Taichung, Taiwan; University of Houston, United States of America

## Abstract

**Background:**

Chronic spontaneous urticaria (CU) is a common skin disorder, with an estimated prevalence of 0.5–1.8% in most populations. Around 30–50% of CU patients have an autoimmune etiology, with autoantibodies (autoAbs) against IgE, FcεRIα, and FcεRII/CD23. Although the *in vivo* autologous serum skin test (ASST) and *in vitro* histamine release/activation assay are the most frequently used screening methods, these two have many limitations and do not directly measure susceptible autoAbs. This study aimed to establish an *in vitro* rapid screening test using recombinant autoantigen FcεRIα(rFcεRIα) to improve the diagnosis of autoimmune urticaria.

**Methods:**

Forty patients with CU and 20 healthy individuals were enrolled. After PCR-based cloning and the production of extracellular fragments of the FcεRIαprotein using the *E. coli* expression system, serum autoAb to rFcεRIαwas evaluated using in-house ELISA and rapid immunodot test.

**Results:**

In ELISA-based detection, 14 out of 20 CU-ASST(+) patients exhibited anti- FcεRIαresponses, whereas five of the 20 CU-ASST(-) and two of the 20 non-CU patients showed autoantibody background in the assay. For the immunodot test, 55% (11/20) of the CU-ASST(+) sera exhibited anti-FcεRIαreactivity. There was no false positive among the CU-ASST(-) and non-CU groups. Using clinical urticaria plus ASST(+) as the gold standard, in-house ELISA had 70% sensitivity, 82.5% specificity, and positive likelihood ratio of 4, while immunodot had 55% sensitivity, 100% specificity, and positive likelihood ratio >55.

**Conclusions:**

This study has developed a rapid immunodot method with high specificity for detecting autoAb to FcεRIαin patients with CU. Preliminary data indicates that this immunodot technique has the potential to be a routine diagnostic assay for autoimmune CU.

## Introduction

Chronic spontaneous urticaria (CU) is one of the most common skin diseases, with an estimated prevalence of 0.5-1.8% [1]–[3]. Though not fatal, chronic urticaria has a profound impact on a patients' quality of life. Patients with CU have a significantly worse quality-of-life than those with allergic asthma and allergic rhinitis [4], and the disease impact is equivalent to that of triple coronary artery diseases [5]. Moreover, although all age groups can be affected, CU is most frequently seen between 20 to 50 years of age, the primary working years of life [6]. Thus, it not only has a great impact on the patient's quality of life, but also significant direct and indirect health costs [7], [8].

The wheal-and-flare symptoms of CU are very much similar to those of acute urticaria triggered by allergens. However, in most of the CU cases, there is no definite identifiable direct external triggering factor [9]. In many CU cases, considerable evidence point to an autoimmune cause. About 12–30% of patients with chronic spontaneous urticaria also have thyroid autoimmunity [10], [11] and a study has shown that IgE against thyroid peroxidase may be involved in the pathogenesis of CU [12]. Moreover, 40–67% of CU patients test positive to autologous serum skin test (ASST) [6], [13], [14], among which the IgG auto-antibodies directly against the α-subunit of the high-affinity IgE receptor (IgG anti- FcεRIα), FcεRII/CD23, or to IgE itself (IgG anti-IgE) have been identified [15]–[18].

Studies show that functional autoantibodies can initiate urticarial eruptions by cross-linking the IgE receptor with the help of C5a, leading to the release of histamine and other mediators from granules of blood basophils and cutaneous mast cells [19]–[21]. Because only skin mast cells express C5a receptor and not lung, uterine, and tonsilar mast cells [22], the requirement of C5a may explain why patients with these autoantibodies often suffer from cutaneous symptoms but not bronchospasms or anaphylaxis. Patients with CU and functional IgG anti- FcεRIαor IgG anti-IgE are reported to have more severe symptoms and refractory prognosis [14], [23]. Thus, it is necessary to identify the subgroup of autoimmune urticaria among CU patients.

The ASST is frequently been used as a surrogate test for screening for the presence of histamine-releasing functional autoantibodies against FcεRI or IgE clinically [13], [24], [25]. However, some studies note that 20–30% of patients with allergic airway disease but without CU and 40–55% of healthy subjects may also show reactivity to autologous sera [26]–[28], leading to arguments regarding the specificity of ASST in diagnosing autoimmune urticaria [27]. Functional assays measuring basophil histamine release or basophil activation are reported to be more reliable in determining patients with autoimmune CU [29]–[34]. However, the assays are technically cumbersome, which limit the routine use of these assays in daily practice.

The present study aimed to establish an *in vitro* rapid screening test using recombinant autoantigen FcεRIα(rFcεRIα) with higher specificity to improve the diagnosis of autoimmune urticaria.

## Materials and Methods

### Patients and Control Subjects

The Institutional Review Board of Taichung Veterans General Hospital approved the study protocol (Protocol No/IRB TCVGH No: C10200) and all of the study participants provided written informed consent. Forty patients with chronic urticaria, defined as recurrent spontaneous wheals lasting <24 hours and occurring at least twice a week for more than 6 weeks, were recruited from the hospital's Allergy Clinic. Subjects younger than 20 years of age were excluded from the study. All of the subjects received an autologous serum skin test (ASST) and the patients were divided into the CU-ASST(+) and CU-ASST(-) groups. Twenty ASST (-) healthy individuals without CU and allergic/autoimmune diseases volunteered and were enrolled as controls. The sera were stored in −70°C till analysis.

### Autologous Serum Skin Test (ASST)

Venous blood (10 ml) were drawn into sterile tubes without clotting accelerator and allowed to clot at room temperature for 30 minutes. Sera were separated by centrifugation at 500 g for 15 min. Autologous serum (50 µl) was injected intradermally into the patient's forearm. Equal volumes of histamine (0.1 mg/ml) and 0.9% sterile saline served as the positive and negative controls, as described previously [13]. Positive ASST was defined as a wheal-and-flare reaction >1.5 mm in size compared to that of the saline control at 30 min.

### PCR-based Cloning of FcεRIα

Oligodeoxynucleotide primers for FcεRIαwere designed according to previously reported sequences (Gene Bank access No. GI: NM_002001.2; NM_001207019). The primers used for FcεRIαwere sense, 5′-*GGATCC* TTAGCAGTCCCTCAGAAACC-3′ and anti-sense, 5′-*AAGCTT* CTTCTCACGCGGAGCTTTTA-3′ (priming regions underlined, *Bam*HI and *Hin*dIII sites in italics). The products of the first-strand cDNA synthesis (0.2 µg) and primers (20 pmol) were mixed in a reaction mixture containing 50 mM KCl, 10 mM Tris-HCl pH 9.0, 1.5 mM MgCl_2_, 0.01% gelatin, 200 µM of each dNTP, and 2 units Platinum Taq DNA polymerase (Invitrogen, Carlsbad, CA, USA) in a Perkin Elmer thermocycler. The target cDNA was isolated from the gel using a BandPrep kit (Genepure, Taichung, Taiwan) and was cloned into a pCR2.1 vector using a TA cloning kit (Invitrogen).

Positive clones were first selected by blue/white screening and sequenced. After digestion of restriction enzymes, recombinant fragments were further sub-cloned into the expression vector pQE30 and transformed into the *E. coli* strain M15 for maximal expression.

### Purification of Recombinant Proteins

Plasmid pQE30 was carried to a stretch of 6 histidine residues located downstream of the secretion signal. A protease recognition sequence was used for tag removal. The his-tag sequence bound to divalent cations (Ni^2+^) immobilized the histidine-binding metal chelation resin and the protein was recovered by elution with imidazole. Clones carrying the pQE30- FcεRIαcDNA were grown under appropriate conditions to stimulate protein production.

The cell lysate was loaded onto a Ni-immobilized His-Bind affinity column (Novagen, Darmstadt, Germany) and was washed with wash buffer (50 mM NaH_2_PO_4_, 300 mM NaCl, and 20 mM imidazole, pH 8.0). The bound proteins were eluted with elution buffer (50 mM NaH_2_PO_4_, 300 mM NaCl, and 250 mM imidazole, pH 8.0) and stored at −20°C. Protein concentration was determined by the Coomassie Brilliant G-250 protein-dye binding method of Bradford (Bio-Rad, Hercules, CA, USA), with bovine serum albumin as standard.

### Detection of Recombinant Proteins by SDS-PAGE and Immunoblotting

Purified recombinant FcεRIαwas suspended in SDS sample buffer containing 5% 2-mercaptoethanol for SDS-PAGE. Protein samples were loaded on a 4% polyacrylamide stacking gel above a 12% separating gel, which was run with discontinuous buffer. After electrophoresis, the gels were fixed and stained with 0.2% Coomassie Brilliant Blue R-250 (Sigma Biochemical, St. Louis, MO, USA). For immunoblotting, gel was transferred electrophoretically by semidry (Bio-Rad) for 30 minutes at 0.8 mA/cm^2^ to nitrocellulose membranes (Millipore). The membranes were blocked with 0.15% casein in PBS for 2 hours and incubated with a 1∶100 dilution of pooled sera from five of CU-ASST(+) and CU-ASST(-) subjects for 1 hour. In immunoblot inhibition, the serum pool from CU-ASST(+) patients was pre-absorbed at room temperature for 2 hours with recombinant FcεRIαprotein at a final concentration of 30 or 100 µg/ml. After washing, a 1∶10000 dilution of goat anti-human IgG HRP-conjugated solution (Zymed) was added for 1 hour and bands were visualized by adding 3-amino-9-ethylcarbazole (AEC) substrate solution (Sigma).

### Enzyme-Linked Immuno-sorbent Assay (ELISA) for Detecting Anti- FcεRIαAutoantibody

Purified recombinant FcεRIαproteins in 0.1 M sodium carbonate, pH 9.6 were used to coat a 96-well plate (Nunc, Denmark) in duplicate and were incubated overnight at 4°C. After the ELISA, the plate was washed 3 times with PBST and then blocked with 0.15% casein for 2 hours at room temperature. After washing, the serum samples were added at a 1∶100 dilution with PBS to each well, which were then incubated for 2 h at room temperature. After washing, peroxidase-conjugated goat anti-human IgG (Zymed, CA, USA) was added to each well (1∶10,000 dilution), which were then incubated for 1 h at room temperature. The reaction was visualized using 2,2′-azino-bis(3-ethylbenzthiazoline-sulfonic acid (ABTS, Sigma) as substrate for 10 min and read at 415 nm with a Sunrise Absorbance Reader (TECAN, Austria).

A standard curve was generated using the polyclonal human IgG (Octagam, Austria) concentrations in ng/ml and its corresponding absorbance values measured at 415 nm by a logistic regression algorithm. The linear region of the standard curve was used to quantify the anti- FcεRIαantibodies in the serum samples.

### Rapid Immunodot for Detecting Anti- FcεRIαAutoantibody

Ten µl aliquots of recombinant FcεRIαproteins (30 µg/ml in PBS) were dotted onto the centers of individual squares made on a nitrocellulose membrane (NC) and air-dried. Unoccupied sites on the NC were blocked with 0.15% casein in PBS (PBSC) for 10 min. After washing, individual patient serum was diluted 1∶4 in PBSC and incubated for 10 min. Bound IgG were detected using peroxidase-conjugated goat anti-human IgG (Zymed). The reaction was developed using a chemiluminescent substrate solution (Applied Biosystem, Bedford, MA, USA) and the signals were recorded by exposure to an X-ray film. Quantitative analysis of the spots was conducted by video densitometer using the software, Kodak Analyzer.

### Statistical Analysis

Statistical significance between means was analyzed using the Mann-Whitney U test. Analyses were performed using the Statistical Package for the Social Science (IBM SPSS version 22.0; International Business Machines Corp, New York, USA). Statistical significance was set at p<0.05.

## Results

### Demographic Data

Patient characteristics and results of laboratory tests were showed in **Table 1**. Twenty ASST(+) and 20 ASST(-) patients were included in this study. In the CU-ASST(+) group, eight were positive for anti-nuclear antibody (ANA) (40%) and two had thyroid autoantibodies (10%). In the CU-ASST(-) group, two had positive ANA (10%) and one had thyroid autoantibodies (5%). There was no significant difference in total IgE levels between the two groups.

**Table 1:** Subject demographics.

Groups	Control	CU-ASST (-)	CU-ASST (+)	*p*
Patient no.	20	20	20	
Male/Female	4/16	10/10	5/15	
Age (range)	45.9 (21–65)	42.2 (21–81)	35.5 (24–53)	
Total IgE (IU/ml)	ND	129.1±16.6	174.8±49.5	
ANA (+/−)	ND	2/18	8/12	NS
ATG (+/−)	ND	1/19	2/18	NS
ATPO (+/−)	ND	0/20	2/18	NS

Abbreviations: ANA, anti-nuclear antibody; ATG, anti-thyroglobulin antibody; ATPO, anti-thyroperoxidase antibody; ND, not done; NS, no statistical significance.

### Cloning and Purification of Recombinant Autoantigen

**Figure 1:**
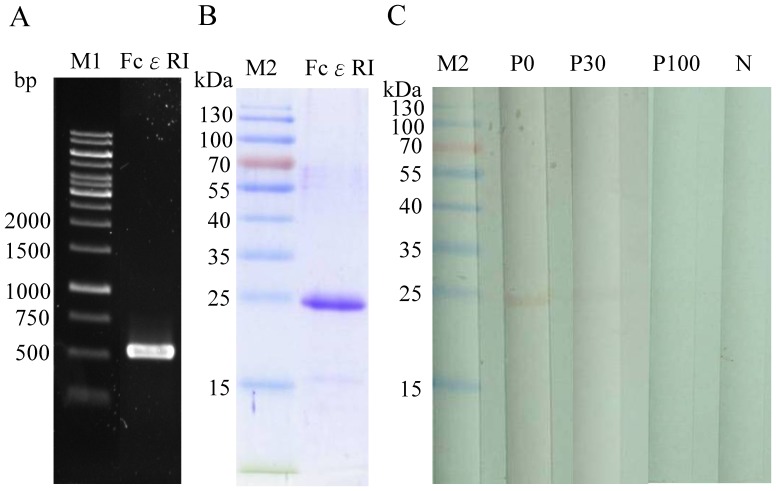
Analysis of cDNA and *E. coli*-expressed protein of FcεRIα. (**A**) Agarose gel electrophoresis of amplified cDNA coding for FcεRIα (534 bp) obtained by RT-PCR from total RNA of human basophils. (**B**) Coomassie blue-stained SDS-PAGE and (**C**) immuno-blotting and immunoblot inhibition of purified recombinant FcεRIα protein with pooled sera from CU-ASST(+) and CU-ASST(-) subjects. Lane P0 was pooled CU-ASST(+) patient sera immunoblotted with purified recombinant FcεRIα protein, lanes P30 and P100 showed pooled CU-ASST(+) sera pre-incubation with 30 or 100 µg/ml of rFcεRIα proteins. Lane N was sera from pooled CU-ASST(-) patients. Numbers on the left indicate sizes of DNA (M1) and protein markers (M2).

The cDNA coding for FcεRIα (**Fig. 1A**) was inserted into the plasmid pQE30 and expressed in *E. coli* M15 as a non-fusion protein. Recombinant FcεRIαprotein was purified using affinity chromatography and analyzed by SDS-PAGE. After electrophoresis, the purified FcεRIαprotein migrated as a single band and displayed >95% purity by Coomassie blue staining (**Fig. 1B**). The IgG-binding specificity of rFcεRIαwas demonstrated (Fig. 1C) and probed with pooled sera from five of CU-ASST(+) and five CU-ASST(-) patients. Pre-incubation of the pooled CU-ASST(+) patient sera with rFcεRIαprotein abolished IgG binding to the 24-kDa rFcεRIα(Fig 1C, lanes P30 and P100).

### ELISA-based Detection of Anti-FcεRIα Autoantibody

The optimum antigen and antibody pair was selected for the standard assay by testing a number of commercially available antibodies and a known quantity of the recombinant protein. A concentration of 0.5 µg/well for coating the FcεRIαrecombinant protein and a dilution of 1/100 of patients' sera were selected for the standard assay. A linear standard curve was constructed using a concentration range (50–800 ng/ml) of polyclonal human IgG in a parallel ELISA. The levels of anti- FcεRIαautoAbs in sera of 20 healthy controls and 40 CU patients by the in-house ELISA were then measured.

**Figure 2:**
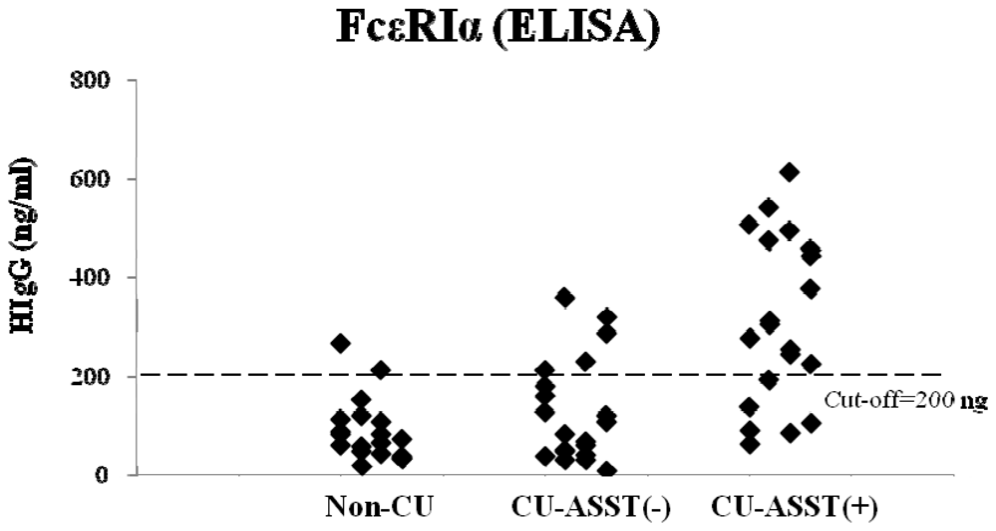
Levels of anti-FcεRIα autoantibody in sera determined by in-house ELISA. Amounts of anti-FcεRIα antibody were calculated using human IgG as a standard. Results were expressed as IgG concentration in serum from individual subjects in all three groups. The horizontal dotted line represented the cut-off value.

The IgG anti-FcεRIαreactivity was expressed as ng/ml. The cut-off value for positive samples was determined as 200 ng, as calculated from the mean plus 2 standard deviations of the data from sera of 20 healthy controls. Thus, 70% (14/20) of CU-ASST(+) patients had specific-IgG levels above the cut-off value of 200 ng/ml IgG in sera (**Fig. 2**). However, 25% (5/20) of CU-ASST(-) patients exhibited anti- FcεRIαreactivity in ELISA, inconsistent with the result of ASST. There was also a 10% (2/20) false positive from the non-CU controls in the assay.

### Dot-based Detection of Anti- FcεRIαAutoantibody

**Figure 3:**
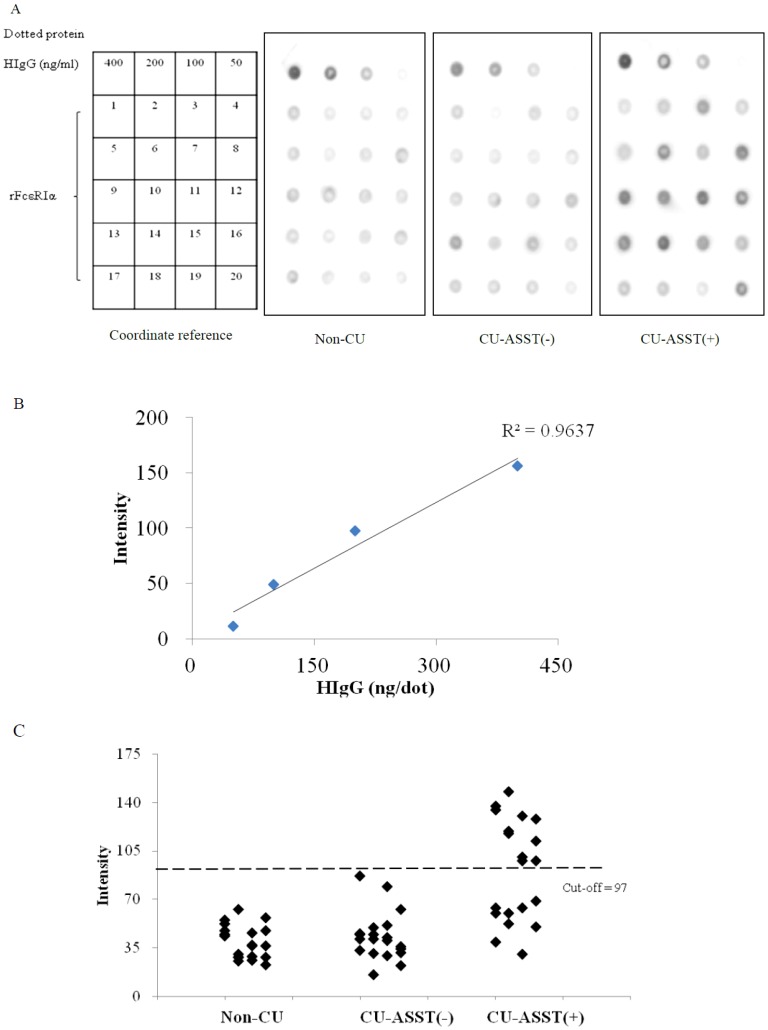
Dot-blot immunoassay of human IgG against recombinant FcεRIα. (**A**) rFcεRIα was tested for reactivity toward serum IgG from 60 tested sera in the indicated groups. The diagram on the left showed the pattern of dotted proteins and coordinated serum number from each group on the blots. (**B**) Correlation curve of the human IgG (HIgG) concentrations and the intensity levels of dot signals by video densitometer. (**C**) Intensity levels of dot signals from (A). The cut-off value was 97 from the mean intensity of 200 ng HIgG, as shown by the dotted line.

To develop a simple and convenient screening assay, an anti- FcεRIαdot test was first evaluated in 60 sera from 20 non-CU healthy controls and 40 CU patients, with human IgG as the standard. The exposure signals on the X-ray films of the 60 tested samples and standard spots ranged from 50 to 400 ng/dot (**Fig. 3A**). There was a statistically significant correlation between IgG concentration and intensity levels of the standard spots (R^2^ = 0.9637, *p*<0.01) (**Fig. 3B**).

After semi-quantitative analysis, the cut-off value for positive samples was determined at an intensity value of 97 estimated from the mean intensity of 200 ng human IgG (HIgG) (**Fig. 3C**). Results obtained from the immunodot revealed that 55% (11/20) of the CU-ASST(+) patients had positive anti- FcεRIαsignals, whereas the CU-ASST(-) and control specimens all showed negative results.

Comparing the ELISA and immunodot methods (**Table 2**), in the ELISA-based detection, 70% (14/20) of CU-ASST(+) patients exhibited anti- FcεRIαresponses, whereas 25% (5/20) of CU-ASST(-) and 10% (2/20) of non-CU controls showed false positive results in the assay. As for the immunodot test, 55% (11/20) of the CU-ASST(+) sera exhibited anti- FcεRIαreactivity. There was no false positive among the CU-ASST(-) and non-CU groups.

**Table 2:** Comparison of two *in vitro* methods for detecting anti-FcεRIα autoantibody in the study*.

Methods	Non-CU	CU-ASST(-)	CU-ASST(+)	sensitivity	specificity	LR+	LR-
ELISA	2/20	5/20	14/20	70%	82.5%	4	0.36
Immunodot	0/20	0/20	11/20	55%	100%	>55	0.45

*Cut-off: 200 ng HIgG.

Using clinical urticaria plus ASST(+) as the gold standard for autoimmune urticaria, the in-house ELISA had 70% sensitivity, 82.5% specificity, and positive likelihood ratio of 4. The immunodot had 55% sensitivity, 100% specificity, and positive likelihood ratio of 55.

### Panel Test of Sera from Chronic Urticaria Subjects for Clinical Application

**Figure 4:**
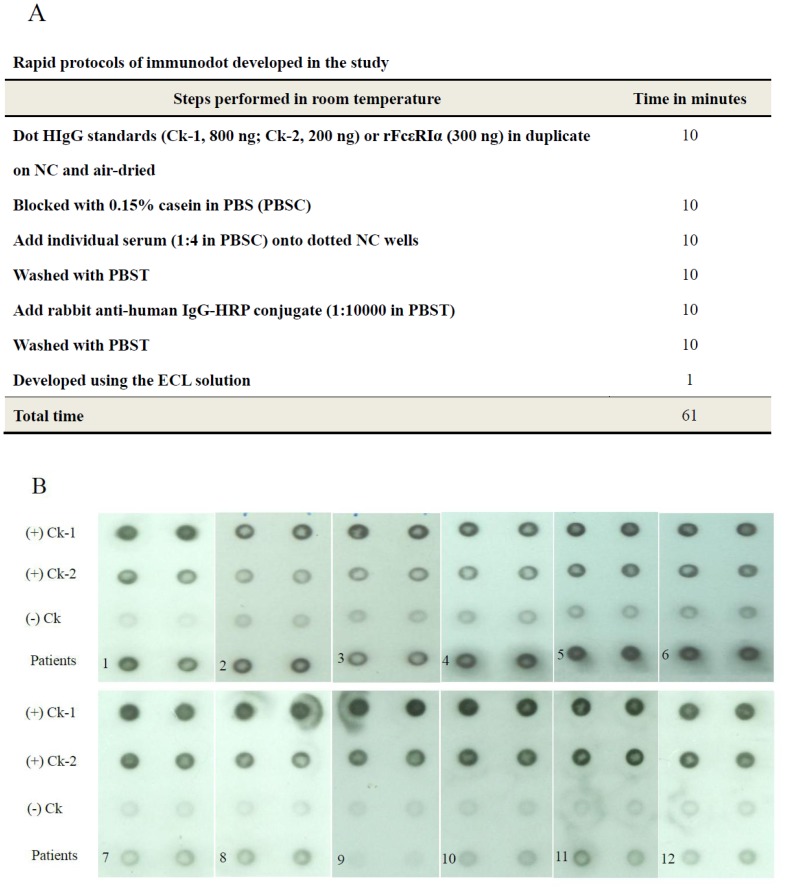
Protocols and experimental results of rapid immunodot. (**A**) Protocols of rapid immunodot for detecting FcεRIα autoantibody in the study. (**B**) Representative results of rapid immunodot with 12 tested sera from CU patients. Patients 1–6 revealed positive responses and patients 7–12 had negative results against rFcεRIα as compared with the positive control-2 (200 ng of HIgG, designated as (+)Ck-2). Positive control-1 using 800 ng of HIgG was designated as (+)Ck-1 and negative control using normal serum was designated as (-)Ck.

To improve convenience in the real world use in the clinics, a rapid panel test was developed. The total time required for the assay completion was 61 min. The rapid immunodot protocol (**Fig. 4A**) and representative results (**Fig. 4B**) revealed that Patients 1–6 had positive responses and Patients 7–12 had negative responses against recombinant FcεRIαprotein defined as signals ≥200 ng HIgG, with the positive control–2 designated as (+)Ck-2. Each panel included normal serum as the negative control, designated as (-)Ck.

## Discussion

It is reported that CU patients with anti- FcεRIαautoantibodies seem to have more severe disease and are more resistant to anti-histamine therapy [23], [35], [36]. Although a recent study suggests that the treatment response to omalizumab may not differ between patients with and those without autoimmune evidences [37], it is still important to differentiate patients with autoantibodies to determine the treatment strategy and predict the prognosis before going into expensive biologic therapy.

The present study evaluates autoAb to extracellular fragments of FcεRIαby in-house ELISA and a rapid immunodot test. The frequency (55–70%) of anti- FcεRIαdetected among CU-ASST(+) patients is similar to previously reported values [16], [38]. Since only one CU-relevant autoantigen, FcεRIα, is used in this study, whether adding more CU-relevant autoantigens like IgE and FcεRII/CD23 in the panel will increase the test sensitivity require further studies. Furthermore, as subjects with allergic airway diseases and atopic dermatitis are not enrolled in this study, whether the result of the immunodot test among this group remain as specific as healthy and CU-ASST(-) controls warrants further investigation.

In the present study, in-house ELISA is performed using the same FcεRIαfragment expressed by *E. coli*, as described by Fiebiger et al. using the baculovirus expression system [38]. The presence of IgG anti-FcεRIα is detected in 70% of 20 CU-ASST(+) patients based on a cut-off defined by non-CU healthy controls. However, there is a 10% false positive reaction in the 20 non-CU-ASST(-) healthy controls by the in-house ELISA. As there is a universal and mandatory DPT vaccination (i.e., diphtheria, tetanus toxoid, and pertusis) policy in Taiwan, all of the subjects enrolled in this study had been vaccinated with tetanus in childhood. One possibility is that anti-FcεRIα autoAbs cross-reacted with tetanus toxoid as reported previously [39].

Comparing the two methods in this study, although in-house ELISA has a higher sensitivity (70% vs. 55%), the specificity of immunodot in detecting anti-FcεRIα IgG is higher than that of in-house ELISA (100% vs. 82.5%). An important advantage of the immunodot test over ELISA is that it requires much less time to obtain the results (1 h vs. 6 h) and it does not require additional equipment.

In conclusion, this study has developed an immunodot method for detecting autoAb to FcεRIα in patients with CU. The method shows high specificity and requires only one hour to obtain the results, without the need for additional equipment. Such preliminary data indicates that the immunodot technique has the potential to be a routine diagnostic assay for autoimmune CU.
